# Fertility navigators in female oncofertility care in an academic medical center: a qualitative evaluation

**DOI:** 10.1007/s00520-020-05412-1

**Published:** 2020-03-20

**Authors:** M. van den Berg, S. Nadesapillai, D. D. M. Braat, R. P. M. G. Hermens, C. C. M. Beerendonk

**Affiliations:** 1 Radboud Institute for Health Sciences (RIHS), Department of Obstetrics and Gynaecology, Radboud University Medical Center, P.O. Box 9101, 6500HB Nijmegen, The Netherlands; 2 Radboud Institute for Health Sciences (RIHS), Department of IQ Healthcare, Radboud University Medical Center, P.O. Box 9101, 6500HB Nijmegen, The Netherlands

**Keywords:** Cancer, Fertility preservation, Oncofertility, Patient navigator, Fertility navigator, Adolescent and young adult

## Abstract

**Purpose:**

To explore patients’ and professionals’ experiences with fertility navigators in female oncofertility care.

**Methods:**

Semi-structured in-depth interviews were conducted with nine female cancer patients and six healthcare professionals to explore their experiences. They were recruited from an academic medical center (referral clinic for female fertility preservation care). Data were analyzed using the concepts of grounded theory.

**Results:**

Patients were satisfied about the supportive role of the fertility navigator in their fertility preservation process: fertility navigators added value as they became “familiar faces” and provided information, emotional support, personal care, and served as patients’ primary contact person. The fertility navigators had a pleasant collaboration with professionals and supported professionals by taking over tasks. To improve the role of fertility navigators, it was suggested that they should always be present in fertility preservation counseling, and attention should be paid to their availability to improve continuity of care.

**Conclusion:**

Fertility navigators provide personal care, improve satisfaction in patients in their oncofertility process, and support professionals. The overview of issues that need to be addressed when assigning fertility navigators in female oncofertility care combined with the improvement suggestions could be used by other centers when considering implementing fertility navigators.

**Electronic supplementary material:**

The online version of this article (10.1007/s00520-020-05412-1) contains supplementary material, which is available to authorized users.

## Introduction

Improvements in the quality of cancer treatment have resulted in higher rates of cancer survival [1, 2]. For this reason, the importance of addressing the late side effects of cancer treatment and long-term quality of life issues has increased [3, 4]. A major quality of life issue for female adolescent and young adult (AYA) cancer patients is the potential loss of fertility. Depending on the type of cancer, fertility can be affected by gonadotoxic treatments like chemotherapy or radiotherapy, or as a consequence of gonadal damage caused by surgery [5]. In order to secure future reproductive function, female AYA cancer patients can undergo a fertility preservation (FP) treatment before the start of their cancer treatment. Current FP options include cryopreservation of embryos, oocytes, ovarian tissue, and ovarian transposition [5].

Studies have shown that female AYA cancer patients would like to be informed about the effects of cancer treatment on their fertility and the FP options available [6–8]. Patients also highlighted the need to obtain this information shortly after the cancer diagnosis and to discuss the FP options with a reproductive specialist to be able to make a well-informed decision in a situation with high time pressure [7–9]. In addition to their information needs, patients indicated that attention should be paid to their emotional needs and personal concerns in FP decision-making [7, 10]. Some patients even report the decision regarding FP the most difficult decision ever made, and almost as distressing as the battle with cancer itself [7, 11, 12]. Unfortunately, patients still report unmet needs, when it comes to personalized care [6, 7, 13].

In cancer care, various navigation programs have been implemented to improve information provision and support within the treatment process [14–16]. Support is provided by patient navigators (PNs), a role usually performed by nurses, social workers, or health educators who are trained for this role [17, 18]. PNs fulfill the role of patient advocates for cancer patients; they provide additional information about medical procedures, refer patients for FP counseling, help patients schedule appointments, coordinate communication among the medical team, and navigate and support patients through the process [14–16, 18, 19].

In recent years, studies have been carried out using PNs in oncofertility care at the oncology department [20, 21]. Initial results indicate that the use of these PNs improves satisfaction in female AYA cancer patients during their oncofertility process [20, 21]. However, patients receive their FP consultation and treatment at the fertility department where the oncology PNs are not available. Regarding FP consultation and decision-making, patients also indicated a need to pay attention to their emotional needs and personal concerns [7].

To meet these needs, we assigned two fertility nurses at our academic medical center fertility department as fertility navigators (FNs) in female oncofertility care as a pilot in October 2016. Our aim is to explore patients’ and healthcare professionals’ experiences with these FNs. In addition, we will explore suggestions for improving FNs’ role to ultimately improve female oncofertility care.

## Methods

In this qualitative study, semi-structured in-depth interviews were conducted with patients and healthcare professionals to explore their experiences with fertility navigators (FNs), and to explore their improvement suggestions. COREQ guidelines were used to report our research.

### Setting and role of fertility navigators

At the Radboud university medical center (Radboudumc), a referral and expertise center for female FP care, FP counseling is performed by gynecologists, specialized in reproductive medicine. They inform female AYA cancer patients about the risk of infertility due to cancer treatment and possible FP options.

In October 2016, two fertility nurses were assigned as FNs at the fertility department to support female AYA cancer patients. Before their assignment, the FNs were trained; first, they visited a patient navigator who worked in a hospital in Belgium to get familiar with the role. Thereafter, they attended numerous FP counseling consultations by the gynecologist to gain experience. After 6 months, they completed this training and fulfilled their role. Their role as FN consisted of the following: the FNs had their first consultation with a patient if a patient chose to undergo a FP treatment after FP counseling with a gynecologist. They provided instructions about hormonal injections, helped patients schedule appointments, performed ultrasound follow-ups, and attended the oocyte collection if possible. Throughout the process, patients could contact the FN if they had any questions or needed support. One week after the oocyte collection, FNs contacted patients by phone to evaluate their condition and answer any remaining questions.

### Participants

Female AYA patients were eligible for participation if they had been diagnosed with cancer, aged 18–40, had undergone FP treatment before their cancer treatment, and had at least one consultation with the FN. They were excluded if FP treatment took place because of a benign disease or recurrent cancer. Patients were randomly recruited in July 2018 by selecting every fifth person on the list of 65 patients cared for by FNs between October 2016 and July 2018 at the Radboudumc. They were approached by a personalized letter from the researchers to participate. To reach data saturation (i.e., the point at which no new information was mentioned), a second round of recruitment was carried out in which every eighth person on the list was randomly selected. All healthcare professionals who perform FP counseling and had worked with the FNs (*N* = 6) were eligible for participation and were invited by e-mail.

### Data collection

To guide interviews, two topic lists were developed; one for patients and one for professionals (supplementary data). These were based on literature and discussions with the research team [6, 7, 17, 22–25]. Patients’ interviews started with explorative questions about their overall experience at the fertility department. This was followed by discussing various topics about FNs’ role, e.g., support, approachability, and guidance through the FP process. Interviews with professionals included questions about the support FNs provided to professionals and professionals’ opinion of navigators’ contribution to patient care. Both the interviews with patients and professionals ended with asking for suggestions to improve FNs’ role. Two pilot interviews were conducted to refine the questions. The interviews were conducted between July and September 2018 by S.N., took place at the Radboudumc or by telephone, depending on patients’ and professionals’ preferences, and lasted approximately 30 min.

### Data analysis

All interviews were audio recorded, transcribed verbatim, and analyzed through grounded theory analysis using the qualitative research software Atlas.ti (version 8.2, Berlin) [26]. Patients’ and professionals’ data were anonymized and analyzed separately. The transcipts were not returned to participants for comments or feedback. The coding process consisted of the following steps. Each step was performed independently by the two authors (M.B. and S.N.) to increase reliability and validity. First, all patients’ and professionals’ interviews were read. Second, both authors selected and labeled phrases describing experiences or improvement suggestions, using open encoding (i.e., using participants’ own words). The descriptive codes that showed resemblance were combined and redefined into specific subthemes. These subthemes were then merged into broader themes by using axial coding. The broader themes formed the conceptual model for patients’ experiences with FNs that was devised by using the grounded theory method. After each step, the results were compared, and any discrepancies were discussed until consensus was reached. In the coding process, obtained data were continuously compared with previous data as is described in the grounded theory method [26]. In addition, each interview was analyzed directly, so new topics could be added to the initial topic lists.

## Results

### Patients’ experiences

**Fig. 1:**
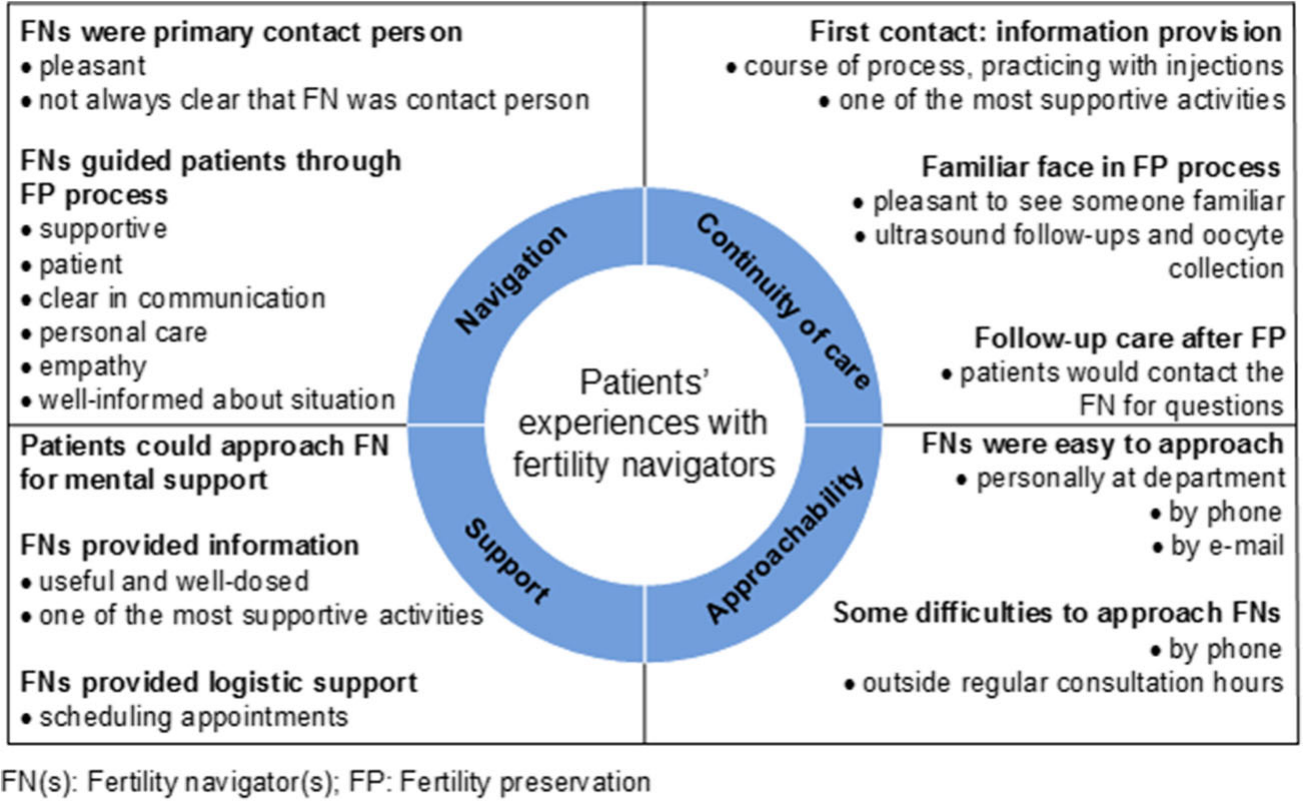
Overview of patients’ experiences with fertility navigators

In the first round of recruitment, 6 out of the 13 selected patients participated, and in the second round, 3 out of the 6 selected patients. The last two patients were interviewed to confirm data saturation. Reasons for declining participation were a lack of time, and some patients did not want to look back on the emotional period. Participants’ characteristics are presented in Table 1. Their experiences with FNs were distributed over four main themes and ten subthemes. An overview of themes and subthemes is presented in Fig. 1 and described in detail below. Illustrative quotes from the interviews are presented in Table 2.

**Table 1:** Demographic patients’ characteristics

Characteristics	Patients, *N* = 9
Mean age, years (range)	32 (20–40)
Level of education^1^	
Low	0
Medium	4
High	5
Marital status during fertility preservation counseling	
Single	3
Partner, but not married	6
Married	0
Type of malignancy	
Breast cancer	7
Hodgkin’s lymphoma	2
Chosen fertility preservation treatment	
Oocyte cryopreservation	6
Embryo cryopreservation	3
First contact with fertility navigator	
January–June 2017	4
July–December 2017	4
January–June 2018	1
Mean time between first contact with fertility navigator and time of interview in months (range)	13 (9–20)

^1^Low, primary school or lower vocational education; medium, secondary or intermediate vocational education; high, higher professional education or university

**Table 2:** Illustrative quotes from patients’ and professionals’ experiences

Themes	Subthemes	Illustrative quotes from patients
Navigation	Primary contact person	“It is just nice to have a primary contact, someone who understands what you are going through, whom you can ask questions to.” (Patient 9) “I know I have had a lot of contact with her [FN], so looking back, yes I guess she was my primary contact, but I cannot remember if that was specifically mentioned.” (Patient 1)
	FNs guided patients through FP process	“It is nice that someone picks you up and literally takes you through the process and, as a figure of speech, drops you off again at the oncology department after two weeks.” (Patient 4) “I really liked that they [FNs] were very personal, for example, they called me by my first name. And they were also really thoughtful, because when you enter this rollercoaster [FP process], it is really nice that they [FNs] not only focus on regulations, but also pay attention to you as a person.” (Patient 2)
Continuity of care	First contact: information provision	“I was still stressed, because I am really afraid of injections, but she really did her best to allay my fears.” (Patient 5) “She also made me inject myself, so I knew what it felt like. That was very pleasant, because, yes you have to inject yourself, and you have to know if you are doing it right.” (Patient 8)
	Familiar face in FP process	“In this process, your body is exposed to everyone, so it’s pleasant that you do not have to repeat your story to someone new when you have to undergo another ultrasound or puncture.” (Patient 2)
	Follow-up care after FP	“I really liked that [telephone contact after FP treatment]. It felt like they [FNs] were still thinking about me and that was a nice feeling.” (Patient 5) “If I would have questions [after cancer treatment], I would approach the fertility navigator and only if she [FN] could not answer, I would approach the doctor, because she [FN] guided and supported me [through the FP process].” (Patient 5)
Support	Patients could approach FN for mental support	“Yes, and I definitely had the feeling that, if I was worried about something, I could approach her [FN].” (Patient 6)
	FNs provided information	“She [FN] provided a lot of information, for example about hormone injections, why those are necessary, what the expected outcome is, but also instructions about the preparation, how to inject yourself, yes she really prepared me for the [FP] process.” (Patient 2)
	FNs provided logistic support	“She [FN] really tried to make it easier for me by combining as much as possible [appointments], so I did not have to come [to the hospital] all the time.” (Patient 9)
Approach ability	FNs were easy to approach	“I received a complete instruction on when to call which person and that went very smoothly. That [instruction] was always given very accurately.” (Patient 7)
	Some difficulties to approach FNs	“I could not call her [FN] directly, but I was also supposed to call the front desk [of the fertility department], so I had problems with reaching her [FN] once. I happen to live close by, so I went there [fertility department] myself.” (Patient 4)
Suggestions for improvement		“Well, I would have liked that one of them [FNs] always performed the ultrasound follow-ups. Because some nurses were not informed about my situation and they [nurses] said [during ultrasound]: Oh you really have a lot of follicles. And I thought, yes, but this is my only chance, and I did not feel like explaining that again.” (Patient 2)
Themes	Subthemes	Illustrative quotes from professionals
Support for professionals	Taking over tasks	“Well, it is really pleasant that I can delegate a lot of tasks to them [FNs], so I can focus on the medical aspect of the [FP] counseling and they [FNs] take care of the practical aspects [of FP treatment].” (Professional 1)
	Back-up	“And what they [FNs] both do... they are well aware of what needs to be done and ask me sometimes: Oh, have you already done this, or did you arrange that?” (Professional 1)
Collaboration with FNs		“They [FNs] try to be very flexible to see [FP] patients, so they are also willing to see patients outside regular consultation hours.” (Professional 2)
	Contact person for other professionals	“For example, other IVF nurses, who have to give an instruction [about hormone injections] to a patient, approach them [FNs] with questions about schedules or medication”. (Professional 5)
	Approachability	“Most of the time I approach them [FNs] in person, I know I can also call them or send an e-mail, but I usually prefer personal contact to discuss what needs to be done.” (Professional 6)
	Availability	“Yes, the availability still deserves attention, certainly. It is just annoying when you do not know if you can count on them [FNs].” (Professional 2)
Suggestions for improvement		“I think a schedule should be made so one of them is always available as fertility navigator.” (Professional 3) “I would prefer that they [FNs] are always present [in FP counseling], so they know exactly what was said, how the patient responded and what subtleties I have made. In addition, the patient also knows that she [FN] has heard it [counseling] and she [patient] can ask questions about the counseling [to the FN].” (Professional 6) “I really think they [FNs] could expand their tasks alongside patient care, they could educate medical students and nurses and eventually [give presentations] on conferences and symposia.” (Professional 3)

FN(s), fertility navigator(s); FP, fertility preservation

### Navigation through the FP process

#### Primary contact person

Most patients mentioned that the FN was their primary contact in the FP process. They knew that they could approach them if they had any questions which were pleasant. However, some patients were unaware that the FN was specifically assigned as their contact person.

#### FNs guided patients through FP process

All patients were satisfied with the FNs’ guidance in their FP process. They mentioned that the FN was very supportive, for example in providing information and reassurance during treatment, was patient, and clear in communication. Furthermore, they indicated that the contact with the FN was pleasant because they were personal and showed empathy. Their personal care was reflected in speaking on first name terms, talking to patients in the waiting room while waiting for appointments, being well-informed about a patient’s personal situation, and in paying attention to you as a person instead of regulations. Moreover, most patients mentioned that the FN took time for them, gave the feeling that they could ask anything, and kept an eye on their FP process.

### Continuity of care

#### First contact: information provision

Most patients had their first contact with the FN after FP counseling with the gynecologist. Patients were informed about hormonal injections and the course of the FP process. Some patients mentioned that practicing with the injections and the reassurance FNs gave while practicing were the most supportive activities.

#### Familiar face in the FP process

Most patients were pleased that the FN, someone familiar, performed ultrasound follow-ups in their process. In some cases, the FN was not available at the follow-up, and although patients understood that they were not always available, others would have preferred them to perform all follow-ups. Furthermore, patients valued the presence of the FN during their oocyte collection and the telephone contact in which they were asked about their condition.

#### Follow-up care after FP

Half of the patients had contact with their FN after the oocyte collection, and they thought that it matched with the personal care they had experienced. The majority mentioned that they would contact the FN again if they had any questions about fertility during or after their cancer treatment.

### Provision of support

#### Patients could approach FN for mental support

None of the patients approached the FN for mental support. However, almost all patients mentioned that they would have approached the FN if they needed mental support because of the personal care that they had experienced. Two patients mentioned that they would have approached the oncology nurse instead of the FN because of the regular follow-ups in and the distance to the hospital where they were being treated for their cancer.

#### FNs provided information

All patients mentioned that the FN provided useful information about FP options, the course of the FP process, expected treatment outcomes, and hormonal injections. In addition, the amount of information was well-dosed given that this was a situation where they had to manage large amounts of information. Many patients noted that providing information about hormonal injections was one of the most supportive activities.

#### FNs provided logistic support

Most patients indicated that the FN helped them schedule FP appointments, taking into account any existing oncology appointments.

### Approachability

#### FNs were easy to approach

Almost all patients mentioned that the FN was easy to approach at the fertility department. The majority asked questions in their follow-up appointments or approached the FN personally at the department. Most patients mentioned that the FN also gave them a card with relevant contact details.

#### Some difficulties to approach FNs

Three out of four patients who contacted the FN by phone were connected directly, while one patient reported having difficulties reaching the FN. Eventually, she had to come to the fertility department to approach the FN personally. Another patient reported that it was difficult to call outside the regular consultation hours.

## Healthcare professionals’ experiences

In total, six professionals had collaborated with the FNs and participated in the interviews. One professional was gynecologist in training and five were gynecologists specialized in reproductive medicine. Professionals’ own experiences with FNs were distributed over two main themes and five subthemes, described in detail below. Illustrative quotes from the interviews are presented in Table 2. Professionals confirmed the following patients’ experiences: FN is a contact person for patients, navigates patients through the FP process, and provides information and mental support.

### Support for professionals

#### Taking over tasks

Almost all doctors reported that the FN provided support by taking over tasks they performed themselves before the implementation of FNs. For example, taking the medical history and entering data in the medical record prior to FP counseling. As a result, the doctor had more time to provide information about FP in the consultation. After this consultation, FNs took over patient care and coordinated planning. One doctor said that she still had to do most tasks by herself, due to limited availability of the FNs.

#### Back-up

A few doctors were pleased that the FNs functioned as a back-up in a process with a lot of arrangements, like scheduling appointments.

### Collaboration with FNs

In general, doctors reported to have a pleasant collaboration. FNs’ qualities that contributed to a pleasant collaboration were as follows: flexibility, dedication, and being well-informed about the entire FP process.

#### Contact person for other professionals

All doctors reported that the FNs were also their primary contact person. Doctors could specifically ask the FN to provide patients additional information after counseling, instead of spending time searching for one of the fertility nurses. Moreover, most doctors mentioned that other fertility nurses also approached the FN as contact person if they had noticed that a patient needed extra support or had questions.

#### Approachability

All doctors mentioned that FNs were easy to approach if they had questions. They preferred to approach them in person at the fertility department instead of by phone or e-mail.

#### Availability

All doctors indicated that FNs’ availability requires further attention. Currently, both FNs work part-time, combining this role with their job as a fertility nurse. As a result, it may happen that both nurses are unavailable as FNs. This leads to a lack of continuity and flow of care in the process for patients, and doctors needed more time to arrange the FP process themselves.

## Suggestions for improvement

Suggestions from patients and professionals to improve FNs’ role are presented in Table 3. Patients mentioned that FNs’ role should be highlighted more in the beginning of the process and that they should always be present in FP counseling, ultrasound follow-up, and oocyte collection. Professionals suggested more improvements, in particular that FNs’ availability and approachability should be improved, that FNs should have a consultation with a patient before FP counseling, and that they should be present in the counseling. Furthermore, their tasks could be expanded when no new FP patients are referred. Illustrative quotes are presented in Table 2.

**Table 3:** Patients’ and professionals’ improvement suggestions

Patients’ suggestions	Professionals’ suggestions
General improvements	General improvements
FNs’ role should be highlighted more in the beginning of the FP process	FNs’ availability should be improved - FNs should have more time as FNs beside their other tasks - Third nurse should be appointed as FN - FNs should be structurally available in regular FP consultation hours
FNs’ approachability should be improved by expanding telephone consultation hours	FN’s approachability should be improved by having their own pager and phone number
Improving FNs’ role in the future	Improving FNs’ role in the future
FNs should always be present in FP counseling	FNs should always have a consultation with the patient prior to FP counseling - To make patients aware of their role - FNs should take a large part of the medical history giving doctors more time to provide information in FP counseling
FNs should perform all ultrasound follow-ups	FNs should always be present in FP counseling but should not perform FP counseling themselves
FNs should be present during oocyte collection	FNs should have contact with other healthcare professionals, particularly oncological caregivers
FNs should be patients’ primary contact person if they start with the IVF-process after recovery of cancer	FNs could support male cancer patients who will undergo semen cryopreservation
	FNs’ tasks could be expanded when no new FP patients are referred, for example: - Taking care of planning regarding ovarian tissue cryopreservation - Completing data in registry retrospectively - Educating students and (oncology) nurses to create awareness about FP

FN(s), fertility navigator(s); FP, fertility preservation

## Discussion

This study explored patients’ and healthcare professionals’ experiences with FNs in female oncofertility care and explored suggestions to improve their role. Patients and professionals were satisfied about the supportive role of the FN. FNs navigated patients through the FP process, improved continuity of care, provided support to patients and professionals, were easy to approach, and collaborated pleasantly. Suggestions to improve their role concerned their presence and availability to further improve continuity of care.

To the best of our knowledge, ours is the first study that describes both patients’ and healthcare professionals’ experiences with FNs at the fertility department in female oncofertility care. Although the Oncofertility Consortium (Chicago, USA) implemented a similar program with a FN [27], they did not describe experiences with this program. Similarities between both programs are the following: the FN was the primary contact person for patients and professionals, navigated patients through the FP process, and provided personal care and information about the course of the process. A difference between the programs was that the FN in Chicago also performed FP counseling, while in our study, gynecologists specialized in reproductive medicine performed the counseling. However, all professionals in our study mentioned that the gynecologist should always perform FP counseling, as they have broader experience and knowledge in complex individual cases. Another difference is that the FN in Chicago was available 24 h a day for FP counseling [27]; it may not be necessary to create a similar 24-h availability in our setting, because none of the patients tried to contact the FNs outside office hours.

In general, our themes and subthemes corresponded with results from previous studies describing patients’ experiences with PNs at an oncology department [20–25]. In these studies, the PN served as patients’ primary contact person at the oncology department guiding them through the cancer treatment process and paying attention to their individual needs [20, 23–25]. In addition, patients in our study were glad that the navigators were aware of their situation and provided personal care. Although previous studies reported that oncology PNs provided emotional support to female cancer patients, we were unable to confirm this in our study. None of our patients approached the FN in their process for mental support. However, patients indicated that they received mental support because the FNs provided personal care, and they would approach them if they would need mental support. Furthermore, as in previous studies, our patients reported that information provision was one of the most supportive activities of the FN [20, 21, 23–25].

A strength of our study is the use of semi-structured in-depth interviews that enabled patients and professionals to mention a variety of important aspects of FNs’ role. In this way, the overall experiences of the two most important groups who had contact with FNs could be explored.

However, several limitations should be considered in the interpretation of our results. Responses might have been influenced by recall bias. Some patients were interviewed more than 1 year after their FP treatment, and they indicated that they did not remember that the FN was specifically assigned to them. Furthermore, most interviewed patients were diagnosed with breast cancer. Patients with other types of cancer may have different experiences with FNs. However, the representation of breast cancer patients in our study can be explained by the relatively high incidence of breast cancer in women of reproductive age [28]. In order to minimize possible bias during analyzing interviews, M.B. and S.N. coded and analyzed all transcriptions separately. Finally, it is uncertain to what extent the implementation of FNs in one single center (the Radboudumc), and their role in female oncofertility care is applicable in other countries, considering the differences in coordination of care and reimbursement.

In the future, more attention should be paid to highlight FNs’ role to patients. Main points to take into consideration in improving their future role are their availability in office hours, their presence in FP counseling, and expanding their tasks alongside patient care. These improvement suggestions combined with our overview of issues that need to be addressed when assigning FNs at a fertility department can be used by other centers when considering implementing FNs.

In conclusion, this study explored patients’ and healthcare professionals’ experiences with FNs in female oncofertility care. They contributed to patient care by navigating patients through the FP process, and providing personal care and information about the process. FNs mainly supported professionals by taking over tasks resulting in more time for them to perform FP counseling. Improvement suggestions can be used to improve FNs’ role at the fertility department to ultimately improve female oncofertility care.

## Electronic supplementary material

ESM 1 (DOCX 14 kb)
