# Supplementary material for: Fertility navigators in female oncofertility care in an academic medical center: a qualitative evaluation
Source: Support Care Cancer. 2020 Mar 20;28(12):5733–41. doi: 10.1007/s00520-020-05412-1 (PMC7686182; doi:10.1007/s00520-020-05412-1)
Supplement: Supplementary file 1 — (DOCX 14 kb) [file 520_2020_5412_MOESM1_ESM.docx]

**Supplementary Data Topic lists for patients and professionals**

**Topic list for patients**

General topics on fertility preservation (FP) process

- Description of course/process at fertility department?
- Experience at fertility department? Positive, negative?
- First contact person?

Specific topics on fertility navigator (FN)

- When was FN introduced? Right moment?
- What did FN do for/with you throughout FP process?
  - Guidance?
  - Support? What? When?
  - Most supportive activity?
- Information provision?
  - Which information?
  - Amount?
- Opportunity to ask questions?
- Communication with FN?
- Approachability?
- When was the last contact with FN? Right moment?
  - Opportunity to ask questions in future?
- Explain role and value of FN in your process?
- Improvement suggestions?
  - Did you miss any form of guidance/support?
  - Ideal role of FN in future?

**Topic list for professionals**

- What is, in your opinion, the role of FNs?
- Any change noticed since introduction FNs? What?
- Advantages of use of FNs? What? Why?
- Disadvantages of use of FNs? What? Why?
- Support?
  - For you? How?
  - For patients? How? Added value?
- Collaboration?
  - Approachability?
  - Contact person?
- Improvement suggestions?
  - What could FN improve? Why? How?
  - Ideal role of FN in future?
